# c-Myc Regulates Proliferation and *Fgf10* Expression in Airway Smooth Muscle after Airway Epithelial Injury in Mouse

**DOI:** 10.1371/journal.pone.0071426

**Published:** 2013-08-13

**Authors:** Thomas Volckaert, Alice Campbell, Stijn De Langhe

**Affiliations:** 1 Department of Pediatrics, Division of Cell Biology, National Jewish Health, Denver, Colorado, United States of America; 2 Department for Molecular Biomedical Research, Unit of Molecular Signal Transduction in Inflammation, VIB, Ghent, Belgium; 3 Department of Biomedical Molecular Biology, Ghent University, Ghent, Belgium; 4 Department of Cellular and Developmental biology, School of Medicine, University of Colorado Denver, Aurora, Colorado, United States of America; University of Giessen Lung Center, Germany

## Abstract

During lung development, Fibroblast growth factor 10 (Fgf10), which is expressed in the distal mesenchyme and regulated by Wnt signaling, acts on the distal epithelial progenitors to maintain them and prevent them from differentiating into proximal (airway) epithelial cells. *Fgf10*-expressing cells in the distal mesenchyme are progenitors for parabronchial smooth muscle cells (PSMCs). After naphthalene, ozone or bleomycin-induced airway epithelial injury, surviving epithelial cells secrete Wnt7b which then activates the PSMC niche to induce *Fgf10* expression. This Fgf10 secreted by the niche then acts on a subset of Clara stem cells to break quiescence, induce proliferation and initiate epithelial repair. Here we show that conditional deletion of the Wnt target gene *c-Myc* from the lung mesenchyme during development does not affect proper epithelial or mesenchymal differentiation. However, in the adult lung we show that after naphthalene-mediated airway epithelial injury c-Myc is important for the activation of the PSMC niche and as such induces proliferation and *Fgf10* expression in PSMCs. Our data indicate that conditional deletion of *c-Myc* from PSMCs inhibits airway epithelial repair, whereas *c-Myc* ablation from Clara cells has no effect on airway epithelial regeneration. These findings may have important implications for understanding the misregulation of lung repair in asthma and COPD.

## Introduction

A complex interplay between endodermal and mesodermal cell types defines early developmental competence and cell fate in the lung. As such, proximal-distal patterning of the lung is accompanied by the gradual restricted ability of developmental progenitors to generate the various epithelial lineages in the mature organ [1]. During lung development, *Fgf10 (Fibroblast growth factor 10)* is expressed in mesenchyme distal to the branching tips where it maintains the multipotent distal epithelial progenitors, but is suppressed proximally and at bifurcation points [2], [3], [4], [5], [6], [7]. We previously identified the *Fgf10*-expressing cells in the distal mesenchyme as parabronchial smooth muscle cell (PSMC) progenitors [3], [8]. *Fgf10* expression as well as the amplification of these PSMC progenitors is regulated by Wnt signaling [3], [9], [10]. Suppression of *Fgf10* expression around the developing airway is crucial to allow for proper maturation of the lung airway epithelium [11], [12], [13], [14], [15].

The adult lung is a vital and complex organ that normally turns over very slowly. The epithelial cells that line the airways are constantly exposed to potential toxic agents and pathogens in the environment, and they must therefore be able to respond quickly and effectively to both cellular damage and local production of immune cytokines. Adult stem cells are implicated in both homeostatic tissue maintenance and functional restoration after injury in organs such as skin and gut.

A widely used lung injury model involves the destruction of Clara cells by naphthalene. Only those Clara cells that express cytochrome P4502F2 (encoded by *Cyp2f2*) are able to convert naphthalene into toxic epoxides leading to cell death. Within a few hours after naphthalene administration nearly all Clara cells have died, except for the few less differentiated variant Clara stem cells that do not express *Cyp2f2*, making them therefore resistant against naphthalene [16], [17], [18], [19], [20]. Ciliated cells quickly spread out, or squamate, under the dying Clara cells in an attempt to cover the basal lamina and maintain the permeability barrier of the epithelium [21].

We have previously shown that surviving ciliated cells after naphthalene, ozone or bleomycin-mediated airway epithelial injury start to secrete Wnt7b, which then activates the PSMC niche to induce *Fgf10* expression [22]. We found that Fgf10 secreted by the niche acts on surviving Clara stem cells to break quiescence, induce proliferation and initiate epithelial repair. Here we show that after naphthalene-mediated airway epithelial injury, the Wnt target c-Myc is important for the activation of the PSMC niche and as such induces proliferation and *Fgf10* expression in PSMCs. Myc proteins coordinate many interdependent processes, including cell growth (increase in cell mass), cell proliferation (DNA replication and cell cycle progression), differentiation and apoptosis [23]. Using an allelic series of mice in which *c-Myc* expression was incrementally reduced to zero, Trumpp et al. showed that fibroblasts from these mice exhibit reduced proliferation and after complete loss of c-Myc function exit the cell cycle [24]. Our data indicate that conditional deletion of *c-Myc* from PSMCs prevents activation of the airway epithelial stem cell niche after airway epithelial injury resulting in deficient epithelial repair.

## Results

### c-Myc Expression in the Lung Mesenchyme is not Required for Normal Lung Development

**Figure 1:**
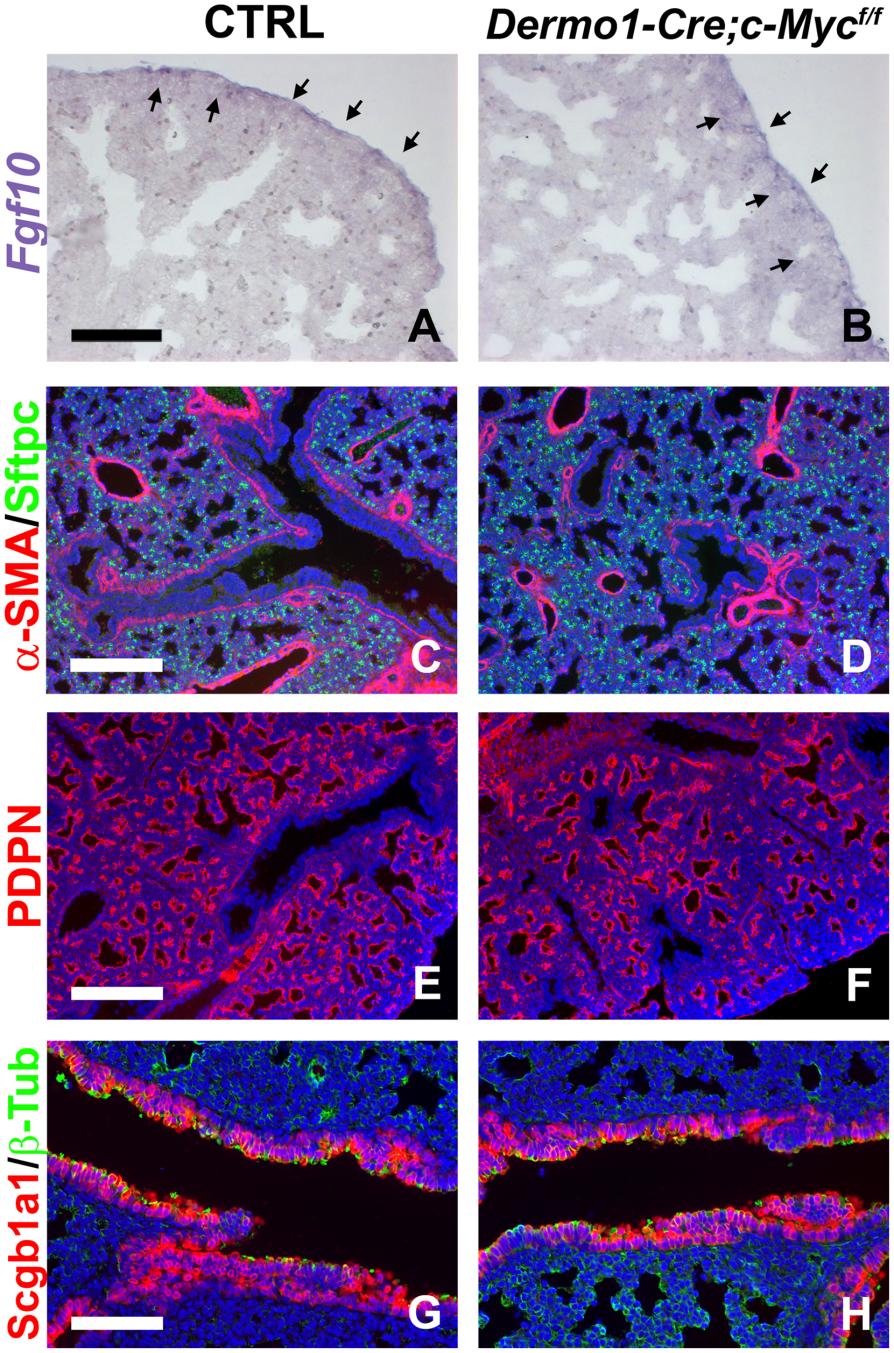
Mesenchyme-specific *c-Myc* ablation does not affect lung development. (A,B) *Fgf10* in situ hybridization on E18.5 ctrl (A) and *Dermo1-Cre;c-Myc^f/f^* (B) lungs showing that *Fgf10* expression is not affected. (C–H) Immunostaining for α-SMA (smooth muscle cells) and Sftpc (ATII cells) (C,D), PDPN (ATI cells) (E,F), and Scgb1a1 (Clara cells) and β-tubulin (ciliated cells) (G,H) on E18.5 ctrl (C,E,G) and *Dermo1-Cre;c-Myc^f/f^* (D,F,H) lungs. n≥3. Scale bars: 100 µM (A,B and G,H); 200 µM (C–F).

During lung development, *Nmyc* expression is normally restricted to a distal population of undifferentiated epithelial cells [25], whereas *c-Myc* is only expressed in the mesenchyme [3]. *c-Myc* expression is regulated by β-catenin signaling and is lost upon conditional deletion of *β-catenin* from the lung mesenchyme [3]. In some organs most of the effects of β-catenin signaling are primarily mediated by *c-Myc* [26]. To test whether during lung development the effects of mesenchymal β-catenin signaling are primarily mediated via *c-Myc* we conditionally deleted *c-Myc* from the lung mesenchyme using a *Dermo1(Twist2)-Cre* line [27]. Interestingly, while ablation of *β-catenin* from the lung mesenchyme resulted in major differentiation defects and reduced *Fgf10* expression [3], [28], we found that conditional deletion of *c-Myc* from the lung mesenchyme has no significant effect on either (Fig. 1A–D). At E18.5, *Dermo1-Cre;c-Myc^f/f^* [24] conditional knock out lungs appear normal, with normal *Fgf10* expression (Fig. 1A,B) and with proper differentiation of the airway and vascular smooth muscle cells (Fig. 1C,D), proper differentiation of the distal epithelium in ATII (Sftpc) and ATI (Pdpn) cells (Fig. 1C–F) and proper differentiation of the bronchial epithelium into Clara (Scgb1a1) and ciliated cells (ß-Tub) (Fig. 1G,H).

### c-Myc Regulates Activation of the Airway Epithelial Stem Cell Niche after Airway Epithelial Injury

**Figure 2:**
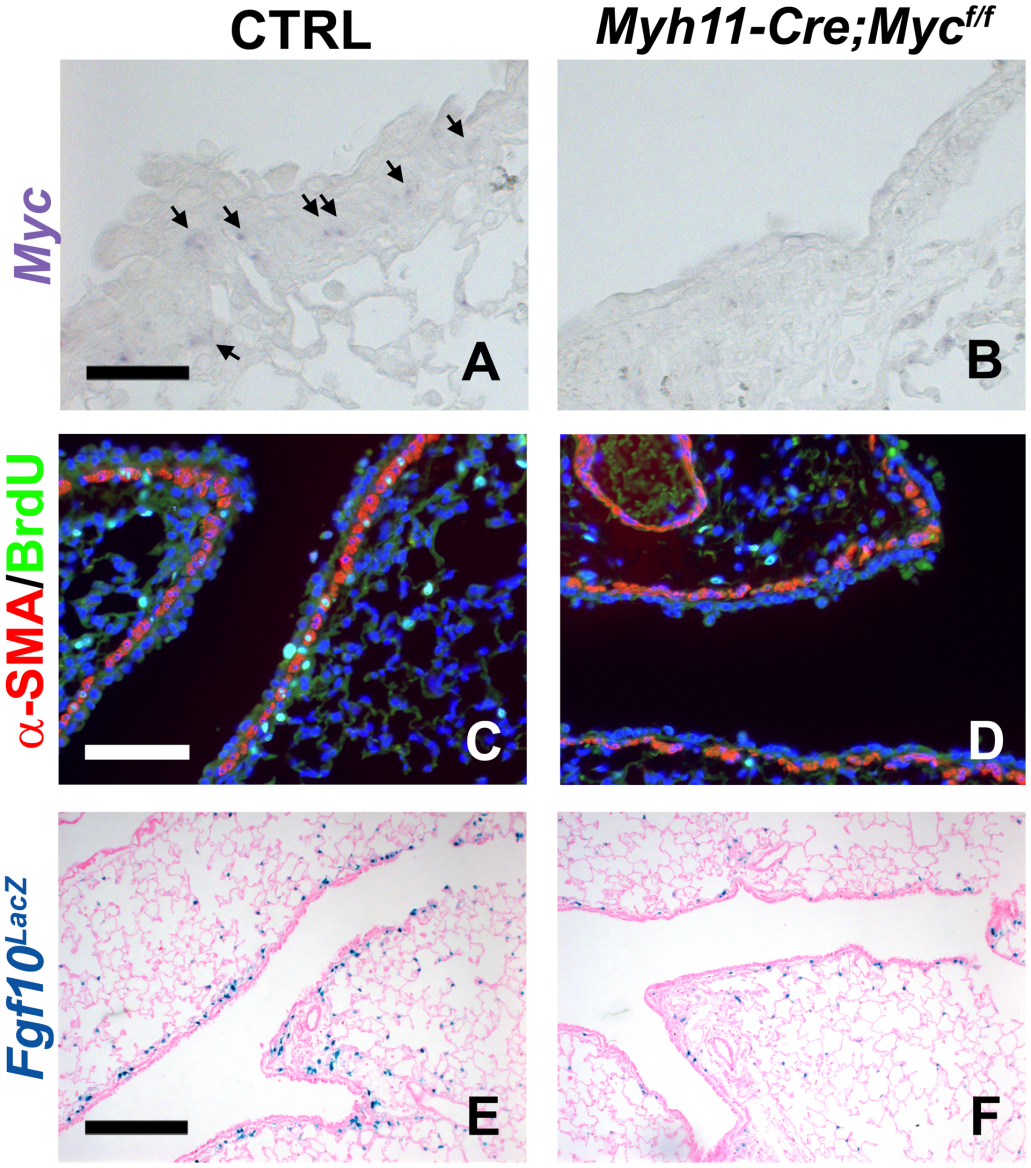
*c-Myc* regulates activation of the airway epithelial stem cell niche after airway epithelial injury. (A,B) *c-Myc* in situ hybridization on ctrl (A) and *Myh11-cre;c-Myc^f/f^* (B) lungs 7 days after naphthalene injury, showing that *c-Myc* is expressed by airway smooth muscle cells after airway epithelial injury and the absence of *c-Myc* expression in *Myh11-cre;c-Myc^f/f^* lungs. (C,D) Immunostaining for BrdU (proliferation marker) and α-SMA (airway smooth muscle cells) on ctrl (C) and *Myh11-cre;c-Myc^f/f^* (D) lungs 3 days after naphthalene treatment. (E,F) β-gal staining on *Fgf10^LacZ^* ctrl (E) and *Myh11-cre;c-Myc^f/f^;Fgf10^LacZ^* (F) lungs 3 days after naphthalene injury. n≥3. Scale bars: 50 µM (A,B); 100 µM (C,D); 200 µM (E,F).

**Figure 3:**
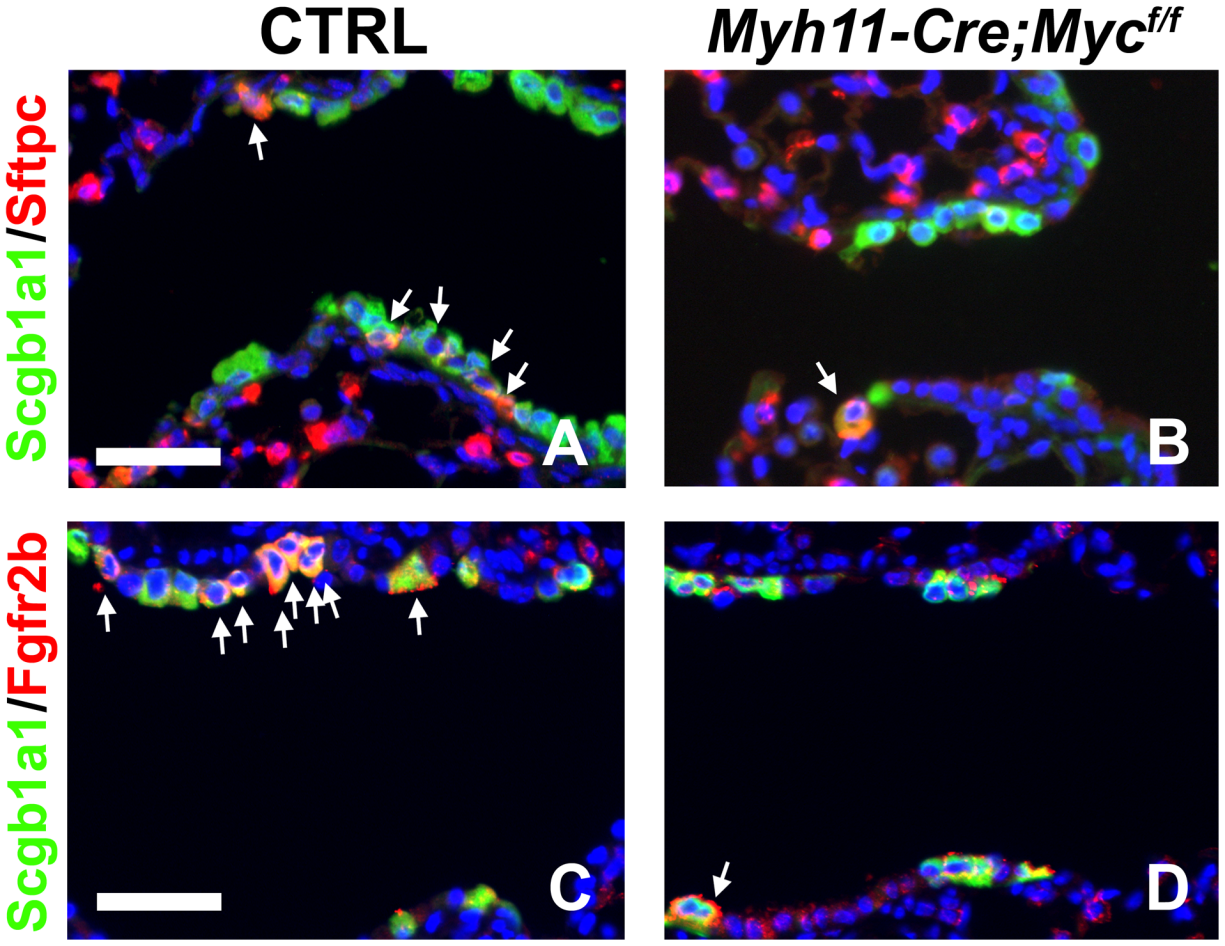
Conditional *c-Myc* deletion from PSMCs reduces Fgf10 signaling in Clara stem cells after naphthalene-mediated injury. (A–D) Immunostaining for Scgb1a1 and Sftpc (A,B), Scgb1a1 and Fgfr2b (C,D) on ctrl (A,C) and *Myh11-cre;c-Myc^f/f^* (B,D) lungs 7 days after naphthalene. n≥3. Scale bars: 50 µM (A–D).

We recently showed that after airway epithelial injury, surviving epithelial cells secrete Wnt7b, which then activates PSMCs (which constitute a niche for airway epithelial stem cells) to induce proliferation and *Fgf10* expression [22]. This Fgf10 secreted by the PSMC niche then acts on a subset of Clara stem cells to break quiescence, induce proliferation and initiate epithelial repair [22]. To investigate the requirement of *c-Myc* in the activation of the PSMC niche and the induction of *Fgf10* expression in the adult lung after airway epithelial injury we generated *Myh11-Cre;c-Myc^f/f^* mice (Myh11: smooth muscle myosin heavy chain) [29], in which we conditionally deleted *c-Myc* from the PSMCs, shown by in situ hybridization in Fig. 2A,B. Interestingly, we found that the PSMC niche in *Myh11-Cre;c-Myc^f/f^* lungs does not get activated after naphthalene-mediated airway epithelial injury. This is manifested by reduced proliferation of the PSMCs, as 9.2% ±1% of PSMCs were BrdU positive in control lungs vs. 2.6% ±0.23% of PSMCs in *Myh11-Cre;c-Myc^f/f^* lungs (*P = *0.000005, n≥4) (Fig. 2C,D) [22]. To investigate whether induction of *Fgf10* expression is also regulated by c-Myc we crossed *Myh11-Cre;c-Myc^f/f^* mice with an *Fgf10^LacZ^* reporter line [3], [7], [8], [22], [30]. In contrast to our observations during lung development we found that in the adult lung, 3 days after naphthalene-mediated airway epithelial injury, *Fgf10* expression in the PSMC niche is regulated by c-Myc, as demonstrated by the lack of induction of *Fgf10* expression in *Myh11-Cre;c-Myc^f/f^;Fgf10^LacZ^* mice (Fig. 2F) compared to control littermates (Fig. 2E). We previously reported a similar drastic reduction in *Fgf10* expression and proliferation in PSMCs, after naphthalene-mediated airway epithelial injury, in mice overexpressing *Dkk1*, a secreted inhibitor of Wnt signaling [22]. To investigate whether epithelial Fgf10 signaling is also reduced we checked for Scgb1a1+Sftcp+ [22], [31] and Scgb1a1+Fgfr2b+ [22] double positive Clara stem cells in the regenerating distal airways near bronchoalveolar duct junctions (BADJs). In accordance with our previously reported results showing that Sftpc and Fgfr2b expression in Clara stem cells is at least in part regulated by Fgf10 [22], we found a reduction in Scgb1a1+Sftcp+ and Scgb1a1+Fgfr2b+ double positive distal airway Clara stem cells at the BADJs in *Myh11-Cre;c-Myc^f/f^* mice 7 days after naphthalene injury compared to control littermates (Fig. 3A–D) [22], [31], [32].

### Conditional Deletion of c-Myc from Airway Smooth Muscle Severely Impairs Airway Epithelial Regeneration After Injury

**Figure 4:**
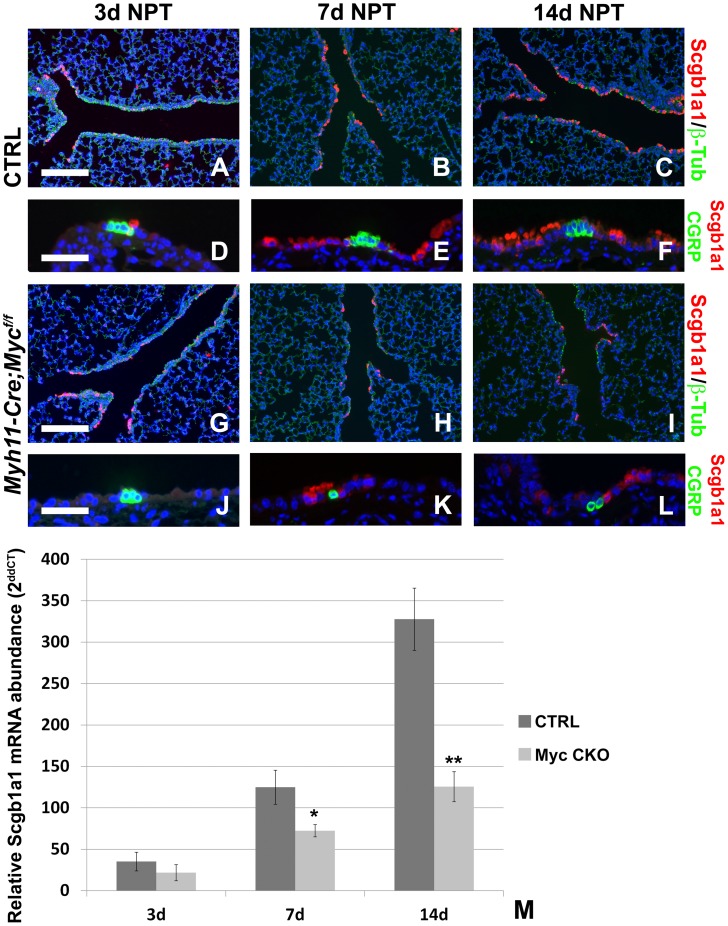
Conditional *c-Myc* deletion from the epithelial stem cell niche impairs epithelial regeneration after injury. (A–L) Immunostaining for Scgb1a1 (Clara stem cells) and β-tubulin (ciliated cells) (A–C,G–I) or Scgb1a1 (Clara stem cells) and CGRP (neuroendocrine bodies) (D–F,J–L) on ctrl (A–F) and *Myh11-cre;c-Myc^f/f^* (G–L) lungs 3 days (A,D,G,J), 7 days (B,E,H,K) and 14 days (C,F,I,L) after naphthalene injury. (M) qPCR analysis of relative *Scgb1a1* mRNA abundance in lungs from ctrl and *Myh11-cre;c-Myc^f/f^* mice 3, 7 and 14 days after naphthalene treatment. **P<0.01, *P<0.05 vs. respective control. n≥3. Scale bars: 200 µM (A–C and G–I); 50 µM (D–F and J–L).

Fgf10 secreted by the PSMC niche after airway epithelial injury is critical for proper regeneration of the airway epithelium [22]. We next investigated how airway epithelial regeneration is affected in *Myh11-Cre;c-Myc^f/f^* mice with a conditional inactivation of *c-Myc* from the PSMC niche. *Myh11-Cre;c-Myc^f/f^* and control littermates were injured with naphthalene resulting in a >95% decrease in *Scgb1a1* expression, as a measure of Clara cell loss, by 3 days after injury and airway epithelial regeneration was monitored over time. At 3 days post injury both control and *Myh11-Cre;c-Myc^f/f^* mice show similar levels of injury demonstrated by low levels of *Scgb1a1* mRNA expression (a Clara stem cell-specific marker) and the presence of limited Scgb1a1 positive Clara stem cells at BADJs and near CGRP-expressing neuroendocrine bodies, while most of the airway is lined with ciliated cells (β-tubulin) (Fig. 4A,D,G,J,M). At 7 days after injury, *Myh11-Cre;c-Myc^f/f^* mice show a 40% decrease in airway epithelial regeneration (Fig. 4H,K,M) compared to control mice (Fig. 4B,E,M). This decrease in regeneration is even more evident at 14 days post injury, with *Myh11-Cre;c-Myc^f/f^* mice showing an almost 3 fold decrease in regeneration (Fig. 4I,L,M) compared to control mice (Fig. 4C,F,M).

### Conditional Deletion of c-Myc from Clara Stem Cells does not Affect Airway Epithelial Regeneration after Injury

**Figure 5:**
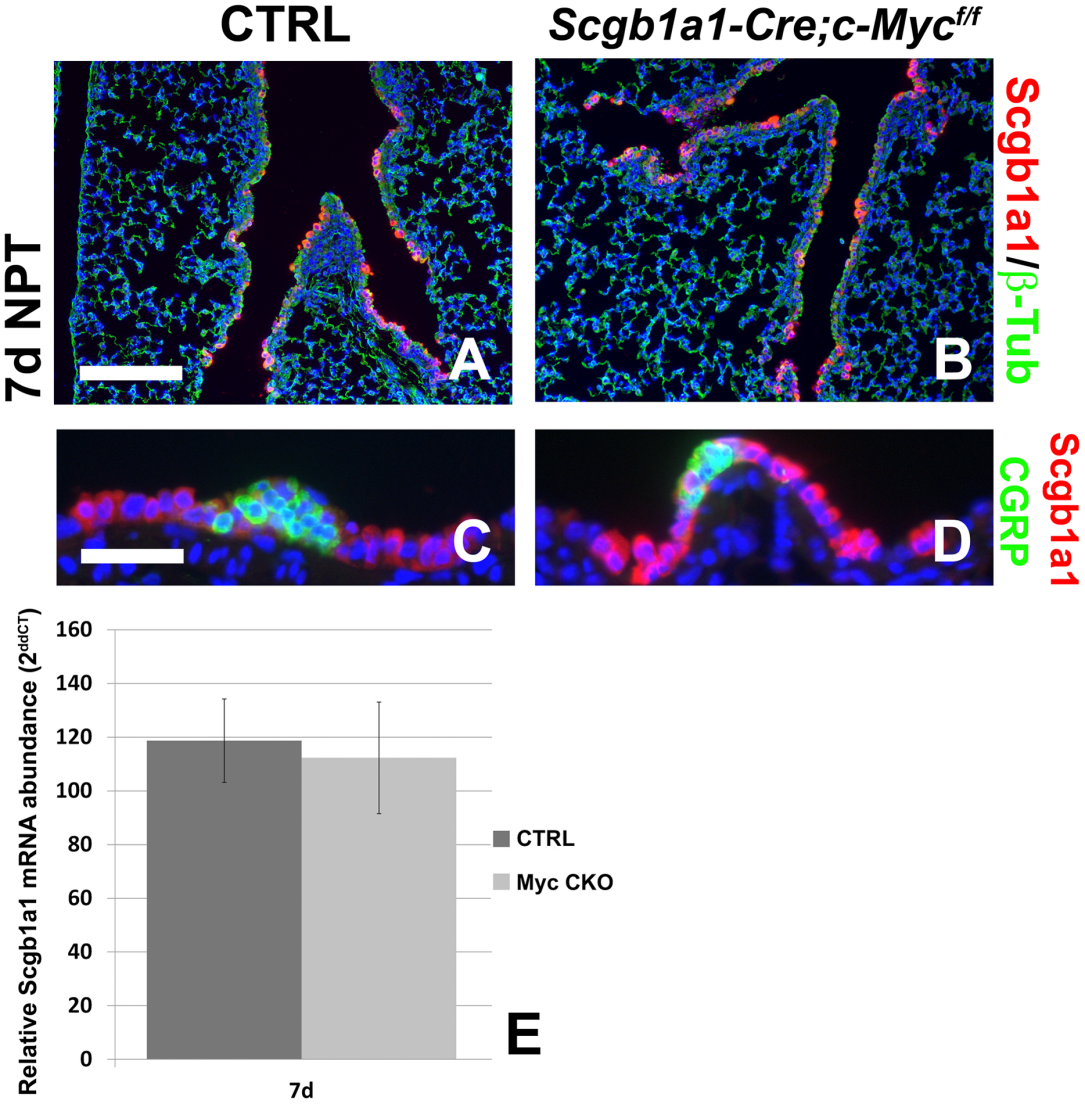
Epithelial *c-Myc* deletion does not affect airway epithelial regeneration after injury. (A–D) Immunostaining for Scgb1a1 (Clara stem cells) and β-tubulin (ciliated cells) (A,B) or Scgb1a1 (Clara stem cells) and CGRP (neuroendocrine bodies) (C,D) on ctrl (A,C) and *Scgb1a1-Cre;c-Myc^f/f^* (B,D) lungs 7 days after naphthalene injury. (E) qPCR analysis of relative *Scgb1a1* mRNA abundance in lungs from ctrl and *Scgb1a1-Cre;c-Myc^f/f^* mice 7 days after naphthalene treatment. *P*>0.05, n≥3. Scale bars: 200 µM (A,B); 50 µM (C,D).

We have previously shown that after naphthalene injury a subset of Clara cells undergo a transient epithelial to mesenchymal transition (EMT) to acquire stem cell-like properties and as such are able to transiently induce the expression of *Myh11* [22]. To investigate whether the decrease in airway epithelial regeneration in *Myh11-Cre;c-Myc^f/f^* mice is not due to deletion of *c-Myc* from these Clara cells, transiently expressing *Myh11-Cre*, we generated *Scgb1a1-Cre;c-Myc^f/f^* [33], [34] mice in which the *c-Myc* gene is deleted specifically from all Clara cells. We found that airway epithelial regeneration after naphthalene injury is not affected in *Scgb1a1-Cre;c-Myc^f/f^* mice compared to control littermates (Fig. 5A–E), indicating that epithelial c-Myc does not play an important role in airway epithelial regeneration and that the defect in regeneration observed in *Myh11-Cre;c-Myc^f/f^* mice can be attributed solely to the loss of *c-Myc* from the PSMC niche. This is consistent with the fact that during lung development *c-Myc* expression is restricted to the mesenchyme, whereas *Nmyc* is expressed solely in the epithelium [3], [25].

## Discussion

The lung has a complex three-dimensional structure that features major differences along its proximal-distal axis in terms of the composition of the endoderm-derived epithelium. The trachea and primary lung buds arise by different morphogenetic processes from contiguous regions of the embryonic foregut [35]. A distinguishing feature of the adult mouse cartilaginous airways (i.e. trachea and primary bronchi) is that *Fgf10* is expressed in the mesenchyme between the cartilage rings [36], [37] and that they contain a discontinuous population of basal stem cells that express p63 and specific keratins (K14 and K5). In addition to basal cells, the luminal epithelium in cartilaginous airways consists of two main columnar epithelial cell types: ciliated cells and Clara cells with a limited number of Clara cell-derived goblet cells. Ciliated cells contain cilia which are involved in the clearance of mucus produced by goblet cells, whereas Clara cells produce secretoglobins, the most abundant of which is Scgb1a1 (also known as CCSP, CC10 and CCA) [38], [39], [40].

The more distal airways (small bronchi and bronchioles) have a columnar epithelium surrounded by airway smooth muscle which does not express *Fgf10* during normal homeostasis [8]. Clara stem cells predominate over ciliated cells and there are more neuroendocrine cells than in the trachea. More importantly, there is no evidence of basal cells in smaller airways in the mouse during normal homeostasis [41].

In the cartilaginous airways basal cells are considered to be on top of the stem cell hierarchy and are able to self renew and give rise to both Clara cells, goblet cells and ciliated cells [42]. Clara cells themselves are also considered stem cells and during normal homeostasis can give rise to new Clara cells and terminally differentiated ciliated cells [43], [44]. Cellular plasticity (including but not limited to differentiation, dedifferentiation, and transdifferentiation) is a frequently encountered cell behavior during injury repair [45], [46], [47], [48], [49], [50], [51]. Interestingly, p63 is a master regulator required for the development of basal cells [52] and induces a basal cell phenotype and squamous metaplasia when ectopically expressed in Clara cells [53]. This form of Clara cell reprogramming may happen to some extent after airway epithelial injury, as under such conditions Clara cells have been shown to be able to give rise to basal cells [44].

Interestingly, our unpublished data suggest that Fgf10 plays a role in the differentiation of airway epithelial cells into basal stem cells during lung development (Volckaert et al., manuscript submitted).

Our data presented here indicate that c-Myc plays an important role in regulating the activity of the PSMC niche in the adult lung. We found a role for c-Myc in regulating proliferation of PSMCs as well as the induction of *Fgf10* expression within PSMCs cells after airway epithelial injury. Interestingly, we found no important role for c-Myc in the mesenchyme during lung development indicating that the function of c-Myc during lung development is redundant and that other not yet identified factors may compensate for the loss of *c-Myc* during lung development. The lack of defective smooth muscle cell differentiation or maintenance in *Myh-Cre;c-Myc^f/f^* lungs suggests that c-Myc may play a specific role in activation of the PSMC niche after injury. Together with the finding that epithelial c-Myc does not play an important role in lung epithelial homeostasis or repair after injury we conclude that targeting c-Myc may be a great way to treat lung diseases characterized by abnormal proliferation of smooth muscle cells, such as asthma and pulmonary arterial hypertension in which Wnt signaling plays a role [54]. In addition, we have previously shown that *Fgf10* secreted by the PSMCs modulates the differentiation of Clara cells into goblet cells [22], which is a hallmark of the asthmatic airway. Future experiments will be needed to determine if loss of mesenchymal *c-Myc* may also reduce proliferation of (myo)fibroblasts in the bleomycin model of pulmonary fibrosis, in which Wnt signaling plays an important role [55], [56], [57], [58], [59], [60], [61]. If so, targeting c-Myc might be an effective and selective way to treat fibroproliferative lung diseases in general.

## Materials and Methods

### Study Approval

All experiments were conducted in strict accordance with the recommendations in the guide for the care and use of laboratory animals. The protocol was approved by the National Jewish Health institutional animal care and use committee #AS2774.

### Mouse Strains

*Myh11-Cre [Tg(Myh11-cre,-EGFP)2Mik/J]* mice were obtained from Jackson Laboratories. *Dermo1-Cre* mice were a kind gift from Dr. David Ornitz [27]. *Scgb1a1-Cre* were a kind gift from Dr. Thomas Mariani [33], [34]. *c-Myc^f/f^* mice were a kind gift from Dr. Andreas Trumpp [24]. Adult mice were 8 weeks old at time of naphthalene administration. Animals were maintained in a pathogen-free environment.

### β-gal Staining

Tissues containing *Fgf10^LacZ^* allele were dissected, and β-gal staining was performed at 3 days after naphthalene injury. Lungs were dissected and fixed in 4% PFA in PBS at room temperature for 5 minutes, rinsed in PBS, injected with freshly prepared X-gal solution, transferred into a vial of X-gal solution, and stained at 37°C overnight. After rinsing with PBS, lungs were postfixed in 4% PFA in PBS at room temperature overnight. For microtome sections, after 4% PFA fixation, lungs were washed in PBS, dehydrated, and paraffin embedded.

### Immunofluorescence

All staining was done on paraffin sections of formalin-fixed lungs. Immunofluorescent staining was performed with the following primary antibodies: mouse anti–β-tubulin (3F3-G2; Seven Hills Bioreagents), goat anti-Scgb1a1 (T-18; Santa Cruz Biotechnology Inc.), rabbit anti-Scgb1a1 (Seven Hills Bioreagents), rabbit anti-CGRP (Sigma-Aldrich), rabbit anti-Fgfr2 (Bek) (C-17; Santa Cruz Biotechnology Inc.), mouse anti–α-SMA cy3 conjugate and unconjugated (14A; Sigma-Aldrich), rabbit anti-Sftpc (Seven Hills Bioreagents), mouse anti-PDPN (Iowa hybridoma bank). All fluorescent staining was performed with secondary antibodies from Jackson Immunoresearch (except the Cy3-conjugated α-SMA) and mounted using Vectashield with DAPI (Vector Labs). Photographs were taken with a Zeiss AxioImager and Axiovision software.

### qPCR

RNA was isolated from lung accessory lobes using RNALater (Ambion) and Total RNA Kit I (Omega Biotek) according to the manufacturer’s instructions. RNA concentration was determined by spectrophotometry. cDNA was generated using SuperScript III First-Strand Synthesis System (Invitrogen) according to the manufacturer’s instructions. Comparative real-time PCR was performed for *β-glucuronidase* (Mm00446953_m1) and *Scgb1a1* (Mm00442046_m1) Taqman Gene Expression Assays (Applied Biosystems) using a StepOne Plus system (Applied Biosystems). *β-glucuronidase* was used as a reference control to normalize equal loading of template cDNA.

### Naphthalene Treatment

Naphthalene (Sigma-Aldrich) was dissolved in corn oil at 30 mg/ml and administered intraperitoneally at 8 weeks of age, with doses adjusted according to strain to achieve a 95% decrease in the abundance of *Scgb1a1* mRNA in total lung RNA of WT mice at 3 days after injection. Control mice for regeneration studies were WT littermates.

### Proliferation

Mice were given intraperitoneal injections of 10 µl BrdU (GE Healthcare) per gram body weight 4 hours before sacrifice. Lungs were fixed in 4% paraformaldehyde, dehydrated, and paraffin embedded. Sections were treated with monoclonal anti-BrdU (clone BU-1; GE Healthcare) according to the manufacturer’s instructions. FITC-labeled anti-mouse secondary antibodies were used (Jackson Immunoresearch). All slides were mounted using Vectashield with DAPI.

### In situ Hybridization

In situ hybridization on paraffin sections of formalin-fixed lungs was performed as previously described [3]. A 584-bp *Fgf10* mouse cDNA [2] and 201-bp fragment of *c-Myc* [3] mouse cDNA were used as templates for the synthesis of digoxigenin-labeled antisense riboprobes.

### Statistics

For BrdU labeling and qPCR analysis, each experiment was repeated with samples obtained from at least 3 different lungs preparations. All results are expressed as mean ± SEM. The significance of differences between 2 sample means was determined by the Student’s t test. *P* values less than 0.05 were considered statistically significant.

## References

[1] Rawlins EL, Clark CP, Xue Y, Hogan BL (2009) The Id2+ distal tip lung epithelium contains individual multipotent embryonic progenitor cells. Development 136: 3741–3745. 19855016 10.1242/dev.037317 PMC2766341

[2] Bellusci S, Grindley J, Emoto H, Itoh N, Hogan BL (1997) Fibroblast growth factor 10 (FGF10) and branching morphogenesis in the embryonic mouse lung. Development 124: 4867. 9428423 10.1242/dev.124.23.4867

[3] De Langhe SP, Carraro G, Tefft D, Li C, Xu X, et al (2008) Formation and differentiation of multiple mesenchymal lineages during lung development is regulated by beta-catenin signaling. PLoS One 3: e1516. 18231602 10.1371/journal.pone.0001516 PMC2211394

[4] De Langhe SP, Carraro G, Warburton D, Hajihosseini MK, Bellusci S (2006) Levels of mesenchymal FGFR2 signaling modulate smooth muscle progenitor cell commitment in the lung. Dev Biol 299: 52–62. 16989802 10.1016/j.ydbio.2006.07.001

[5] Goss AM, Tian Y, Cheng L, Yang J, Zhou D, et al.. (2011) Wnt2 signaling is necessary and sufficient to activate the airway smooth muscle program in the lung by regulating myocardin/Mrtf-B and Fgf10 expression. Dev Biol. 10.1016/j.ydbio.2011.06.011 PMC3319016 21704027

[6] Nyeng P, Norgaard GA, Kobberup S, Jensen J (2008) FGF10 maintains distal lung bud epithelium and excessive signaling leads to progenitor state arrest, distalization, and goblet cell metaplasia. BMC Dev Biol 8: 2. 18186922 10.1186/1471-213X-8-2 PMC2263027

[7] Ramasamy SK, Mailleux AA, Gupte VV, Mata F, Sala FG, et al (2007) Fgf10 dosage is critical for the amplification of epithelial cell progenitors and for the formation of multiple mesenchymal lineages during lung development. Dev Biol 307: 237–247. 17560563 10.1016/j.ydbio.2007.04.033 PMC3714306

[8] Mailleux AA, Kelly R, Veltmaat JM, De Langhe SP, Zaffran S, et al (2005) Fgf10 expression identifies parabronchial smooth muscle cell progenitors and is required for their entry into the smooth muscle cell lineage. Development 132: 2157–2166. 15800000 10.1242/dev.01795

[9] Chen F, Cao Y, Qian J, Shao F, Niederreither K, et al (2010) A retinoic acid-dependent network in the foregut controls formation of the mouse lung primordium. J Clin Invest 120: 2040–2048. 20484817 10.1172/JCI40253 PMC2877937

[10] Goss AM, Tian Y, Tsukiyama T, Cohen ED, Zhou D, et al (2009) Wnt2/2b and beta-catenin signaling are necessary and sufficient to specify lung progenitors in the foregut. Dev Cell 17: 290–298. 19686689 10.1016/j.devcel.2009.06.005 PMC2763331

[11] Sedita J, Izvolsky K, Cardoso WV (2004) Differential expression of heparan sulfate 6-O-sulfotransferase isoforms in the mouse embryo suggests distinctive roles during organogenesis. Dev Dyn 231: 782–794. 15499561 10.1002/dvdy.20173

[12] Izvolsky KI, Shoykhet D, Yang Y, Yu Q, Nugent MA, et al (2003) Heparan sulfate-FGF10 interactions during lung morphogenesis. Dev Biol 258: 185–200. 12781692 10.1016/s0012-1606(03)00114-3

[13] McKeehan WL, Wang F, Kan M (1998) The heparan sulfate-fibroblast growth factor family: diversity of structure and function. Prog Nucleic Acid Res Mol Biol 59 135–76: 135. 10.1016/s0079-6603(08)61031-4 9427842

[14] Izvolsky KI, Zhong L, Wei L, Yu Q, Nugent MA, et al (2003) Heparan sulfates expressed in the distal lung are required for Fgf10 binding to the epithelium and for airway branching. Am J Physiol Lung Cell Mol Physiol 285: L838–846. 12818887 10.1152/ajplung.00081.2003

[15] Shimokawa K, Kimura-Yoshida C, Nagai N, Mukai K, Matsubara K, et al (2011) Cell surface heparan sulfate chains regulate local reception of FGF signaling in the mouse embryo. Dev Cell 21: 257–272. 21839920 10.1016/j.devcel.2011.06.027

[16] Giangreco A, Reynolds SD, Stripp BR (2002) Terminal bronchioles harbor a unique airway stem cell population that localizes to the bronchoalveolar duct junction. Am J Pathol 161: 173–182. 12107102 10.1016/S0002-9440(10)64169-7 PMC1850682

[17] Hong KU, Reynolds SD, Giangreco A, Hurley CM, Stripp BR (2001) Clara cell secretory protein-expressing cells of the airway neuroepithelial body microenvironment include a label-retaining subset and are critical for epithelial renewal after progenitor cell depletion. Am J Respir Cell Mol Biol 24: 671–681. 11415931 10.1165/ajrcmb.24.6.4498

[18] Plopper CG, Macklin J, Nishio SJ, Hyde DM, Buckpitt AR (1992) Relationship of cytochrome P-450 activity to Clara cell cytotoxicity. III. Morphometric comparison of changes in the epithelial populations of terminal bronchioles and lobar bronchi in mice, hamsters, and rats after parenteral administration of naphthalene. Lab Invest 67: 553–565. 1434534

[19] Reynolds SD, Giangreco A, Power JH, Stripp BR (2000) Neuroepithelial bodies of pulmonary airways serve as a reservoir of progenitor cells capable of epithelial regeneration. Am J Pathol 156: 269–278. 10623675 10.1016/S0002-9440(10)64727-X PMC1868636

[20] Stripp BR, Maxson K, Mera R, Singh G (1995) Plasticity of airway cell proliferation and gene expression after acute naphthalene injury. Am J Physiol 269: L791–799. 8572241 10.1152/ajplung.1995.269.6.L791

[21] Park KS, Wells JM, Zorn AM, Wert SE, Laubach VE, et al (2006) Transdifferentiation of ciliated cells during repair of the respiratory epithelium. Am J Respir Cell Mol Biol 34: 151–157. 16239640 10.1165/rcmb.2005-0332OC PMC2644179

[22] Volckaert T, Dill E, Campbell A, Tiozzo C, Majka S, et al (2011) Parabronchial smooth muscle constitutes an airway epithelial stem cell niche in the mouse lung after injury. J Clin Invest 121: 4409–4419. 21985786 10.1172/JCI58097 PMC3204843

[23] Dang CV (2012) MYC on the path to cancer. Cell 149: 22–35. 22464321 10.1016/j.cell.2012.03.003 PMC3345192

[24] Trumpp A, Refaeli Y, Oskarsson T, Gasser S, Murphy M, et al (2001) c-Myc regulates mammalian body size by controlling cell number but not cell size. Nature 414: 768–773. 11742404 10.1038/414768a

[25] Okubo T, Knoepfler PS, Eisenman RN, Hogan BL (2005) Nmyc plays an essential role during lung development as a dosage-sensitive regulator of progenitor cell proliferation and differentiation. Development 132: 1363–1374. 15716345 10.1242/dev.01678

[26] Sansom OJ, Meniel VS, Muncan V, Phesse TJ, Wilkins JA, et al (2007) Myc deletion rescues Apc deficiency in the small intestine. Nature 446: 676–679. 17377531 10.1038/nature05674

[27] Yu K, Xu J, Liu Z, Sosic D, Shao J, et al (2003) Conditional inactivation of FGF receptor 2 reveals an essential role for FGF signaling in the regulation of osteoblast function and bone growth. Development 130: 3063–3074. 12756187 10.1242/dev.00491

[28] Yin Y, White AC, Huh SH, Hilton MJ, Kanazawa H, et al (2008) An FGF-WNT gene regulatory network controls lung mesenchyme development. Dev Biol 319: 426–436. 18533146 10.1016/j.ydbio.2008.04.009 PMC2757945

[29] Xin HB, Deng KY, Rishniw M, Ji G, Kotlikoff MI (2002) Smooth muscle expression of Cre recombinase and eGFP in transgenic mice. Physiol Genomics 10: 211–215. 12209023 10.1152/physiolgenomics.00054.2002

[30] Kelly RG, Brown NA, Buckingham ME (2001) The arterial pole of the mouse heart forms from Fgf10-expressing cells in pharyngeal mesoderm. Dev Cell 1: 435–440. 11702954 10.1016/s1534-5807(01)00040-5

[31] Kim CF, Jackson EL, Woolfenden AE, Lawrence S, Babar I, et al (2005) Identification of bronchioalveolar stem cells in normal lung and lung cancer. Cell 121: 823–835. 15960971 10.1016/j.cell.2005.03.032

[32] Chen H, Matsumoto K, Brockway BL, Rackley CR, Liang J, et al (2012) Airway epithelial progenitors are region specific and show differential responses to bleomycin-induced lung injury. Stem Cells 30: 1948–1960. 22696116 10.1002/stem.1150 PMC4083019

[33] Ji H, Houghton AM, Mariani TJ, Perera S, Kim CB, et al (2006) K-ras activation generates an inflammatory response in lung tumors. Oncogene 25: 2105–2112. 16288213 10.1038/sj.onc.1209237

[34] Simon DM, Arikan MC, Srisuma S, Bhattacharya S, Andalcio T, et al (2006) Epithelial cell PPARgamma is an endogenous regulator of normal lung maturation and maintenance. Proc Am Thorac Soc 3: 510–511. 10.1513/pats.200603-034MS 16921131

[35] Cardoso WV, Lu J (2006) Regulation of early lung morphogenesis: questions, facts and controversies. Development 133: 1611–1624. 16613830 10.1242/dev.02310

[36] Sala FG, Del Moral PM, Tiozzo C, Alam DA, Warburton D, et al (2011) FGF10 controls the patterning of the tracheal cartilage rings via Shh. Development 138: 273–282. 21148187 10.1242/dev.051680 PMC3005603

[37] Tiozzo C, De Langhe S, Carraro G, Alam DA, Nagy A, et al (2009) Fibroblast growth factor 10 plays a causative role in the tracheal cartilage defects in a mouse model of Apert syndrome. Pediatr Res 66: 386–390. 19581825 10.1203/PDR.0b013e3181b45580 PMC3725279

[38] Rawlins EL, Hogan BL (2006) Epithelial stem cells of the lung: privileged few or opportunities for many? Development 133: 2455–2465. 16735479 10.1242/dev.02407

[39] Rock JR, Hogan BL (2011) Epithelial progenitor cells in lung development, maintenance, repair, and disease. Annu Rev Cell Dev Biol 27: 493–512. 21639799 10.1146/annurev-cellbio-100109-104040

[40] Rock JR, Randell SH, Hogan BL (2010) Airway basal stem cells: a perspective on their roles in epithelial homeostasis and remodeling. Dis Model Mech 3: 545–556. 20699479 10.1242/dmm.006031 PMC2931533

[41] Pack RJ, Al-Ugaily LH, Morris G (1981) The cells of the tracheobronchial epithelium of the mouse: a quantitative light and electron microscope study. J Anat 132: 71–84. 7275793 PMC1233396

[42] Rock JR, Onaitis MW, Rawlins EL, Lu Y, Clark CP, et al (2009) Basal cells as stem cells of the mouse trachea and human airway epithelium. Proc Natl Acad Sci U S A 106: 12771–12775. 19625615 10.1073/pnas.0906850106 PMC2714281

[43] Evans MJ, Johnson LV, Stephens RJ, Freeman G (1976) Renewal of the terminal bronchiolar epithelium in the rat following exposure to NO2 or O3. Lab Invest 35: 246–257. 957607

[44] Rawlins EL, Okubo T, Xue Y, Brass DM, Auten RL, et al (2009) The role of Scgb1a1+ Clara cells in the long-term maintenance and repair of lung airway, but not alveolar, epithelium. Cell Stem Cell 4: 525–534. 19497281 10.1016/j.stem.2009.04.002 PMC2730729

[45] Barroca V, Lassalle B, Coureuil M, Louis JP, Le Page F, et al (2009) Mouse differentiating spermatogonia can generate germinal stem cells in vivo. Nat Cell Biol 11: 190–196. 19098901 10.1038/ncb1826

[46] Bedada FB, Gunther S, Kubin T, Braun T (2006) Differentiation versus plasticity: fixing the fate of undetermined adult stem cells. Cell Cycle 5: 223–226. 16397414 10.4161/cc.5.3.2364

[47] Cobaleda C, Busslinger M (2008) Developmental plasticity of lymphocytes. Curr Opin Immunol 20: 139–148. 18472258 10.1016/j.coi.2008.03.017

[48] Kai T, Spradling A (2004) Differentiating germ cells can revert into functional stem cells in Drosophila melanogaster ovaries. Nature 428: 564–569. 15024390 10.1038/nature02436

[49] Stocum DL (2004) Tissue restoration through regenerative biology and medicine. Adv Anat Embryol Cell Biol 176: III–VIII, 1–101, back cover. 10.1007/978-3-642-18928-9 15079897

[50] Stocum DL (2004) Amphibian regeneration and stem cells. Curr Top Microbiol Immunol 280: 1–70. 14594207 10.1007/978-3-642-18846-6_1

[51] Zhou Q, Brown J, Kanarek A, Rajagopal J, Melton DA (2008) In vivo reprogramming of adult pancreatic exocrine cells to beta-cells. Nature 455: 627–632. 18754011 10.1038/nature07314 PMC9011918

[52] Daniely Y, Liao G, Dixon D, Linnoila RI, Lori A, et al (2004) Critical role of p63 in the development of a normal esophageal and tracheobronchial epithelium. Am J Physiol Cell Physiol 287: C171–181. 15189821 10.1152/ajpcell.00226.2003

[53] Koster MI, Kim S, Mills AA, DeMayo FJ, Roop DR (2004) p63 is the molecular switch for initiation of an epithelial stratification program. Genes Dev 18: 126–131. 14729569 10.1101/gad.1165104 PMC324418

[54] Cohen ED, Ihida-Stansbury K, Lu MM, Panettieri RA, Jones PL, et al (2009) Wnt signaling regulates smooth muscle precursor development in the mouse lung via a tenascin C/PDGFR pathway. J Clin Invest 119: 2538–2549. 19690384 10.1172/JCI38079 PMC2735923

[55] Konigshoff M, Balsara N, Pfaff EM, Kramer M, Chrobak I, et al (2008) Functional Wnt signaling is increased in idiopathic pulmonary fibrosis. PLoS One 3: e2142. 18478089 10.1371/journal.pone.0002142 PMC2374879

[56] Konigshoff M, Eickelberg O (2009) WNT signaling in lung disease: a failure or a regeneration signal? Am J Respir Cell Mol Biol 42: 21–31. 19329555 10.1165/rcmb.2008-0485TR

[57] Konigshoff M, Kramer M, Balsara N, Wilhelm J, Amarie OV, et al (2009) WNT1-inducible signaling protein-1 mediates pulmonary fibrosis in mice and is upregulated in humans with idiopathic pulmonary fibrosis. J Clin Invest 119: 772–787. 19287097 10.1172/JCI33950 PMC2662540

[58] Xi Y, Wei Y, Sennino B, Ulsamer A, Kwan I, et al. Identification of pY654-beta-catenin as a critical co-factor in hypoxia-inducible factor-1alpha signaling and tumor responses to hypoxia. Oncogene. 10.1038/onc.2012.530 PMC3871884 23246962

[59] Ulsamer A, Wei Y, Kim KK, Tan K, Wheeler S, et al. Axin pathway activity regulates in vivo pY654-beta-catenin accumulation and pulmonary fibrosis. J Biol Chem 287: 5164–5172. 22203675 10.1074/jbc.M111.322123 PMC3281604

[60] Kim Y, Kugler MC, Wei Y, Kim KK, Li X, et al (2009) Integrin alpha3beta1-dependent beta-catenin phosphorylation links epithelial Smad signaling to cell contacts. J Cell Biol 184: 309–322. 19171760 10.1083/jcb.200806067 PMC2654298

[61] Kim KK, Wei Y, Szekeres C, Kugler MC, Wolters PJ, et al (2009) Epithelial cell alpha3beta1 integrin links beta-catenin and Smad signaling to promote myofibroblast formation and pulmonary fibrosis. J Clin Invest 119: 213–224. 19104148 10.1172/JCI36940 PMC2613463

